# MK2 Is Required for Neutrophil-Derived ROS Production and Inflammatory Bowel Disease

**DOI:** 10.3389/fmed.2020.00207

**Published:** 2020-06-12

**Authors:** Tao Zhang, Junhang Jiang, Jingting Liu, Lu Xu, Shixin Duan, Lei Sun, Wenjuan Zhao, Feng Qian

**Affiliations:** ^1^Engineering Research Center of Cell and Therapeutic Antibody, Ministry of Education, School of Pharmacy, Shanghai Jiao Tong University, Shanghai, China; ^2^Shanghai Pharmaceuticals Holding Co. Ltd., Shanghai, China; ^3^Anhui Province Key Laboratory of Translational Cancer Research, Bengbu Medical College, Bengbu, China

**Keywords:** MAPK-activated protein kinase 2 (MK2), reactive oxygen species, neutrophils, NADPH oxidase, inflammatory bowel disease (IBD)

## Abstract

Inflammatory bowel disease (IBD) is a chronic disease that is commonly accompanied by increased inflammatory responses and elevated reactive oxygen species (ROS) of the gastrointestinal tract. Here, we found that MAPK-activated protein kinase 2 (MK2) modulates ROS production and is required for dextran sulfate sodium (DSS)-induced IBD in the mouse model. Genetic ablation of MK2 in the myeloid lineage cells (MK2^Lyz2−KO^) protected against DSS-induced colitis injury. In response to DSS challenge, compared to MK2^lyz2−WT^ mice, MK2^Lyz2−KO^ mice exhibited less damage of epithelial and goblet cells, decreased generation of interleukin (IL)-6, tumor necrosis factor (TNF)-α, and ROS, as well as reduced Ki67-positive cells and concentrations of myeloperoxidase (MPO) in the intestinal epithelium. Furthermore, upon treatment with formylated peptide N-formyl-methionyl-leucyl-phenylalanine (fMLF), the generation of ROS was attenuated in MK2-deficient neutrophils, in which the phosphorylation of Akt and p38 MAPK was also reduced. Collectively, these findings indicated that MK2 is required for neutrophil-derived ROS production and IBD, and MK2 and ROS are promising therapeutic targets for IBD.

## Introduction

Inflammatory bowel diseases (IBDs), including Crohn's disease (CD) and ulcerative colitis (UC), are characterized by chronic uncontrolled intestinal inflammation (1). The patients with IBD display multiple symptoms such as weight loss, abdominal pain, recurrent diarrhea, and bleeding (2). It impacts on the quality of the patient's life and causes a great challenge to health care systems (3). Over the past half century, the incidence and prevalence of IBD showed a fast increasing tendency worldwide (4, 5). In recent years, some progress has been made to treat IBD, but IBD remains an incurable and awful clinical problem. Thus, it is necessary to comprehensively understand underlying mechanisms and potential treatment targets for IBD.

IBD is a complex clinical disease which arises as a result of the interaction of genetic and environmental factors leading to immune responses and inflammation in the intestines (6). The characteristic histological phenomenon in IBD is an influx of innate immune cells [macrophages, neutrophils, dendritic cells, and natural killer (NK) cells] as well as adaptive immune cells (T cells and B cells) into the lamina (7). During the process of IBD, activated immune cells secrete amounts of inflammatory cytokines including tumor necrosis factor (TNF)-α, interleukin (IL)-1β,IL-6, and reactive oxygen species (ROS) (1, 8). Accumulating data from both experimental animal models and clinical trials indicate that neutrophils, oxidative stress, and pro-inflammatory cytokines are all involved in the development of IBD (9, 10).

Neutrophils play an important role in the pathogenesis of IBD (11). When intestinal epithelium is injured, neutrophils are first activated by chemokines and recruited to the site of infection. They can recognize, phagocytose, and kill pathogens by producing ROS by releasing lytic enzymes from granules (10). On the other hand, the accumulation of activated neutrophils further damages the epithelium and destroys the integrity of the barrier by releasing ROS, proteinases, and cationic peptides (12). Oxidative stress has been proved to be involved in the pathogenesis of IBD, and ROS plays a vital role in damaging intestinal tissues (13–15). However, the molecular mechanisms that regulate neutrophil-derived ROS in IBD remain poorly understood and need to be further explored.

MAPK-activated protein kinase 2 (MK2) is a member of the serine-/threonine-protein kinase family (16). Phosphorylated by p38 MAPK, MK2 is known to be involved in many cellular processes including stress and inflammatory responses, nuclear export, gene expression regulation, and cell proliferation (17). MK2, as a pro-inflammatory factor, played a key role in series diseases, such as rheumatoid arthritis (RA), psoriasis, vasculitis, and IBD (18, 19). Recently, Wang et al. (20) reported that MK2 inhibitor, MMI-0100, could ameliorate dextran sulfate sodium (DSS)-induced colitis in mice, suggesting that MK2 might be a therapeutic target for IBD. However, the specific role of MK2 and its way of action in the pathogenesis of IBD are still unclear and need to be further explored. In the present study, we used MK2^Lyz2−KO^ mice, a mouse strain with MK2 conditional deficiency in myeloid lineage cells, to identify whether the inhibition targeting MK2 in myeloid lineage cells could ameliorate IBD and affect neutrophils releasing ROS and to explore the potential mechanisms.

In the present study, we found that MK2 conditional deficiency in myeloid lineage cells markedly alleviated colon damage and inflammation and inflammatory reaction in a mouse IBD model. In response to DSS challenge, compared to MK2^lyz2−WT^ mice, MK2^Lyz2−KO^ mice exhibited less damages of epithelium and goblet cells, lower concentration of IL-6, TNF-α, myeloperoxidase (MPO), and ROS, and enhanced capability of cell proliferation. We also determined that MK2 in neutrophils was required for the production of neutrophil-derived ROS. Furthermore, upon treatment with **N-**formyl-methionyl-leucyl**-**phenylalanine (fMLF), the generation of ROS was attenuated in MK2 deficient neutrophils, in which the phosphorylation of Akt and p38 MAPK was also reduced. Collectively, these findings indicated that MK2 was required for neutrophil-derived ROS production and IBD, and MK2 and ROS were promising therapeutic targets for IBD.

## Materials and Methods

### Reagents

fMLF and isoluminol were purchased from Sigma-Aldrich (St. Louis, MO, USA). Antibodies including Phosphor-Akt, Akt, Phosphor-p38 MAPK, p38 MAPK, and β-actin antibodies were ordered from Cell Signaling Technology (Danvers, MA, USA). Ki67 antibody was purchased from Beyotime Institute of Biotechnology (Shanghai, China). ROS assay kits were purchased from Nanjing Jiancheng Bioengineering Institute (Nanjing, China).

### Mice

MK2^loxP/loxP^ mice were purchased from The Jackson Laboratory (Sacramento, CA). Lyz2-cre knock-in mice were purchased from the model animal research center of Nanjing University (Nanjing, China). MK2^Lyz2−KO^ mice were generated by mating Lyz2-Cre mice with MK2^loxP/loxP^ mice. All mice used in the study were on C57BL/6 background and 7–10 weeks of age. Mice were kept in a climate-controlled room (25°C, 55% humidity, and 12-h light/darkness cycles), and all procedures were conducted with the use of protocols approved by the Institutional Animal Care and Use Committee at Shanghai Jiao Tong University.

### Colitis Induction With Dextran Sulfate Sodium

DSS-induced colitis model was established using a method described previously (21). Briefly, MK2^Lyz2−KO^ and MK2^Lyz2−WT^ mice (*n* = 6 per group) were given 3.5% DSS (36–50 kd; MP Biomedicals, Solon, OH, USA) in drinking water for 7 days to induce experimental colitis and sacrificed on day 8. During the treatment of DSS, body weight was weighed every day. After sacrificing the mice, the colon length was measured from the end of the cecum to the anus.

### Histology and Immunohistochemistry

For histology studies, colons were removed, fixed in 4% polyoxymethylene overnight, paraffin-embedded, and sectioned. Tissue sections were stained with hematoxylin and eosin (H&E; Beyotime Institute of Biotechnology, China) for microscopic examination and evaluated the tissue injury and inflammation. Epithelial barrier injury score was 0 = normal morphology, 1 = loss of goblet cell, 2 = loss of goblet cells in large areas, 3 = loss of crypts, and 4 = loss of crypts in large areas. Infiltration of leukocyte score was 0 = no infiltrate, 1 = infiltrate around crypt bases, 2 = infiltrate reaching to muscularis mucosae, 3 = extensive infiltration reaching the muscularis mucosae and thickening of the mucosae with abundant edema, and 4 = infiltration to the submucosa. The severity of tissue injury and inflammation was analyzed in a blinded manner.

For immunohistochemistry, after dewaxing and rehydration, the sections were soaked in sodium citrate buffer for heat-induced epitope retrieval and incubated with 10% goat serum for 1 h to block the non-specific binding sites. Then, sections were incubated with anti-Ki67 antibody overnight at 4°C, followed by incubation with horseradish peroxidase (HRP) secondary antibodies for 20 min. The sections were developed by using a diaminobenzidine substrate kit (Boster, China) and counterstained with hematoxylin. Images were obtained with an Olympus BX41 microscope. Ki67-positive cells were counted in five different areas of the section and at least five sections of each mouse.

### Immunostaining of Mucins and Goblet Cells

Mice colon was fixed in 4% polyoxymethylene overnight. Tissues were embedded in paraffin and cut into 5-μm sections. Tissue sections ultimately were dewaxed and stained with Alcian blue/Nuclear Fast Red (Solarbio, China). Briefly, tissue sections was incubated in 3% acetic acid for 3 min and stained in 1% Alcian blue solution for 30–60 min and subsequently stained in 0.1% Nuclear Fast Red for 10–20 min. All operations are performed in room temperature. AB-PAS^+^ goblet cells were counted in five different areas of the section and at least five sections of each mouse.

### Inflammatory Cytokine Measurements

The concentrations of TNF-α and IL-6 in supernatants from colon tissues of mice were evaluated by ELISA according to the manufacturer's instruction (R&D Systems, Minneapolis, MN, USA).

### Colon Tissues Reactive Oxygen Species Determination

The ROS level of the colon tissue was detected using the redox-sensitive fluorescent dye DCFH-DA. Briefly, the frozen colon samples were cut into 5-μm-thick sections and were incubated with DCFH-DA (10 μM/L), which was diluted with phosphate buffered saline (PBS) at 37°C for 30 min, and then washed three times with PBS. Images of the relative level of fluorescent product were captured using a fluorescence microscope connected to an imaging system (TCS SP8, Leica Microsystems). ImageJ software was used to analyze the mean fluorescence intensity of DCFH-DA, which indirectly detected the level of ROS. DCFH-DA-positive areas were analyzed in five different areas of the section and at least five sections of each mouse.

### Colon Tissues Myeloperoxidase Activity

MPO activity in colon tissues (a quantitative measurement of neutrophil infiltration) was assayed as previously described (22). Prepare o-dianisidine dihydrochloride (o-dianisidine) solution by combining o-dianisidine dihydrochloride, ddH_2_O, and potassium phosphate buffer. This solution should be prepared fresh for every assay. Add tissue homogenate in triplicate into a 96-well plate and add diluted H_2_O_2_ to the o-dianisidine mixture. Use a multichannel pipette to add o-dianisidine mixture containing H_2_O_2_ to each of the wells. Measure absorbance at 450 nm using a spectrophotometer. The results were shown as activity units per milligram colon tissues.

### Cell Isolation and Superoxide Production Assays

Mouse polymorphonuclear neutrophils (PMNs) were gathered from bone marrow cell suspensions as described previously (23). Briefly, isolated neutrophils were incubated with 10 μM isoluminol at 37°C for 30 min, and HRP was added to a final concentration of 40 U/ml. Cells were then seeded into a 96-well, flat-bottom tissue culture dish (E&K Scientific). Chemiluminescence was measured every minute using a Wallac multilabel counter plate reader (PerkinElmer, Houston, TX, USA) starting from 5 min before and continuing to 30 min after stimulation with 10 μM fMLP. Unstimulated controls were recorded simultaneously.

### Western Blot

The neutrophils after treatment were washed with PBS and lysed with loading buffer containing protease inhibitor cocktail (Sigma-Aldrich, St. Louis, MO, United States) and phosphatase inhibitor cocktail (Roche Applied Science, Indianapolis, IN, USA). Samples were separated on 10% sodium dodecyl sulfate–polyacrylamide gel electrophoresis (SDS-PAGE) and transferred onto nitrocellulose membranes. The blots were incubated with indicated primary antibodies overnight at 4°C and followed by conjugated secondary antibodies. The blots were detected by ChemiDoc MP System (Bio-Red, USA). The protein band intensities were normalized to β-actin. The intensity was quantified by ImageJ software.

## Statistical Analysis

Each experiment was performed independently for at least three times. The results are presented as the mean ± SEM. Unpaired Student's *t-*test was used for two-group comparison, and one-way *post hoc* test was used for sample analysis with more than two groups. Statistical significance was defined as ^*^P < 0.05, ^**^P < 0.01, and ^***^P < 0.001. Analysis and graphing were performed using the Prism software (ver. 5.0; GraphPad, San Diego, CA).

## Results

### MAPK-Activated Protein Kinase 2 in Myeloid Lineage Cells Contributes to Inflammatory Bowe Disease Induced by Dextran Sulfate Sodium

**Figure 1:**
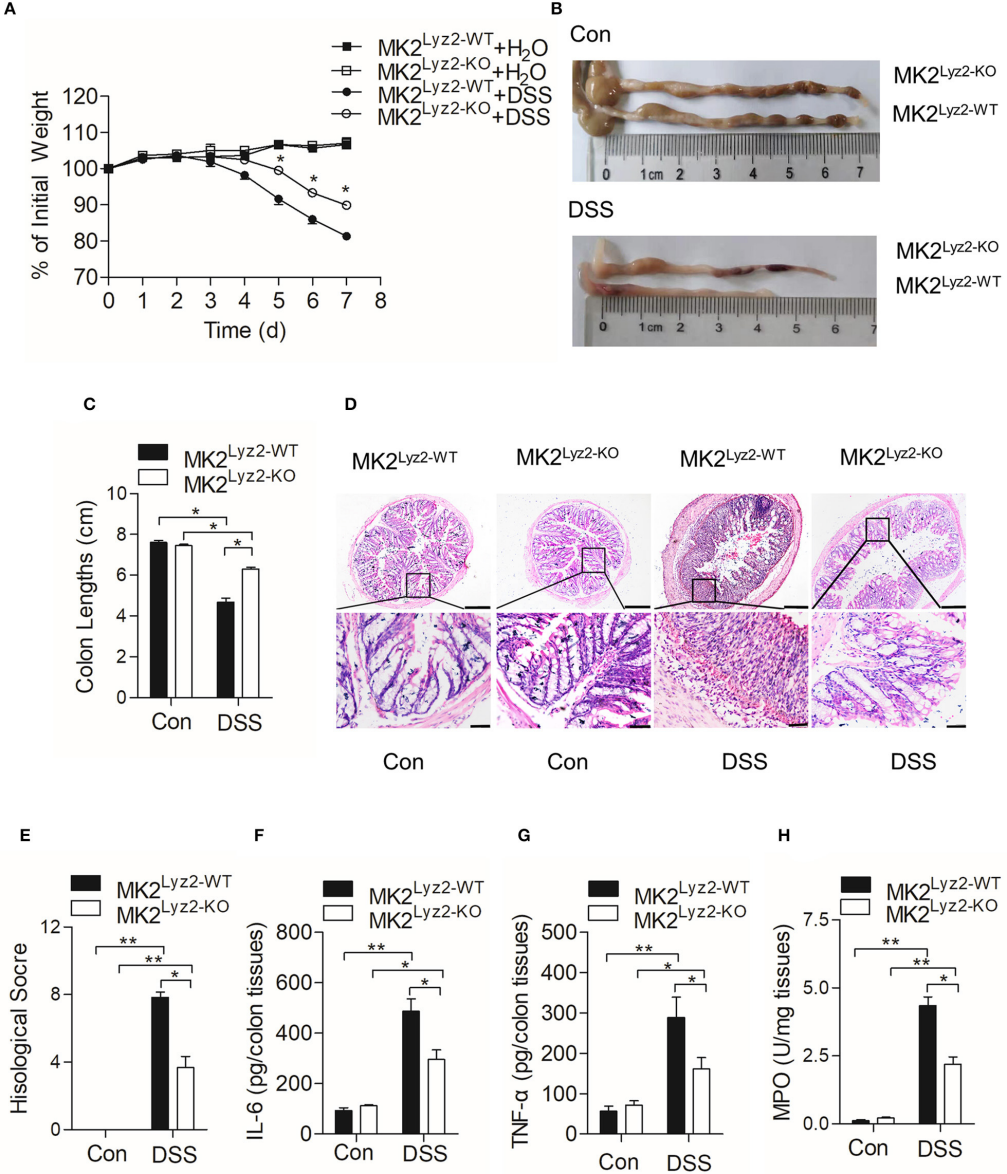
Dextran sulfate sodium (DSS)-induced pathological changes in MK2^lyz2−WT^ and MK2^lyz2−KO^ mice. Male and female mice (7–10 weeks) were used in the experiments. **(A)** The body weight changes of MK2^lyz2−WT^ and MK2^lyz2−KO^ mice receiving 3.5% DSS or water control for up to 7 days. **(B)** Macroscopic examination of colon lengths of MK2^lyz2−WT^ and MK2^lyz2−KO^ mice after 7 days' treatment of 3.5% DSS or water control. **(C)** Statistical analysis of colon lengths. **(D)** Representative images of H&E-stained colon sections from control and DSS-treated mice. Scale bars: 200 μm (upper), 40 μm (lower). **(E)** Statistical analysis of histological scores. **(F)** The protein levels of interleukin (IL)-6 in colon tissues detected by ELISA. **(G)** The protein levels of tumor necrosis factor (TNF)-α in colon tissues detected by ELISA. **(H)** Myeloperoxidase (MPO) activity in the colon tissues. The results were shown as means ± SEM. *P < 0.05; **P < 0.01 based on six mice in each group.

MK2 plays an essential role in the progression of inflammation (24, 25). Wang et al. (20) reported that MK2 inhibitor MMI-0100 could ameliorate DSS-induced colitis in mice through targeting the MK2 pathway. In order to determine the role of myeloid lineage cell MK2 in IBD, we compared pathological changes of DSS-induced IBD in MK2^lyz2−KO^ mice with those in MK2^lyz2−WT^ mice. As shown in Figure 1A, no obvious weight changes were seen in the first 3 days of DSS treatment in both MK2^lyz2−WT^ and MK2^lyz2−KO^ mice. However, starting from fourth day, both MK2^lyz2−WT^ and MK2^lyz2−KO^ mice showed gradual weight reduction, and MK2^lyz2−KO^ mice showed markedly relieved weight reduction compared to MK2^lyz2−WT^ mice during the fifth to seventh days. Consistently, the average colon length of DSS-treated MK2^lyz2−KO^ mice did not obviously shorten as that in MK2^lyz2−WT^ mice (Figures 1B,C). Histological analysis of H&E-stained colonic tissue also showed that MK2^lyz2−KO^ mice exhibited less damage of epithelium compared to the MK2^lyz2−WT^ mice, which showed a complete loss of crypts and a large number of inflammatory cell infiltration in colon tissue with much higher histological scores after 7 days of DSS treatment (Figures 1D,E). The protein level of inflammatory cytokines IL-6 and TNF-α in colon tissues of DSS-treated MK2^lyz2−KO^ mice also did not rise dramatically compared to those of MK2^lyz2−WT^ mice (Figures 1F,G). In addition, to further characterize the extent of inflammation in DSS-treated mice, we observed the activity of MPO and found a lower MPO activity in colon tissues of DSS-treated MK2^lyz2−KO^ mice, suggesting less severe neutrophil infiltration (Figure 1H). Collectively, these data showed that MK2 conditional deficiency in myeloid lineage cells significantly ameliorated pathologic changes of IBD mice induced by DSS with respect to weight loss, colon length, pathological scores, and inflammatory cytokine production.

### MAPK-Activated Protein Kinase 2 in Myeloid Lineage Cells Contributes to Dextran Sulfate Sodium-Induced Goblet Cell Damage and Inhibits Cell Proliferation

**Figure 2:**
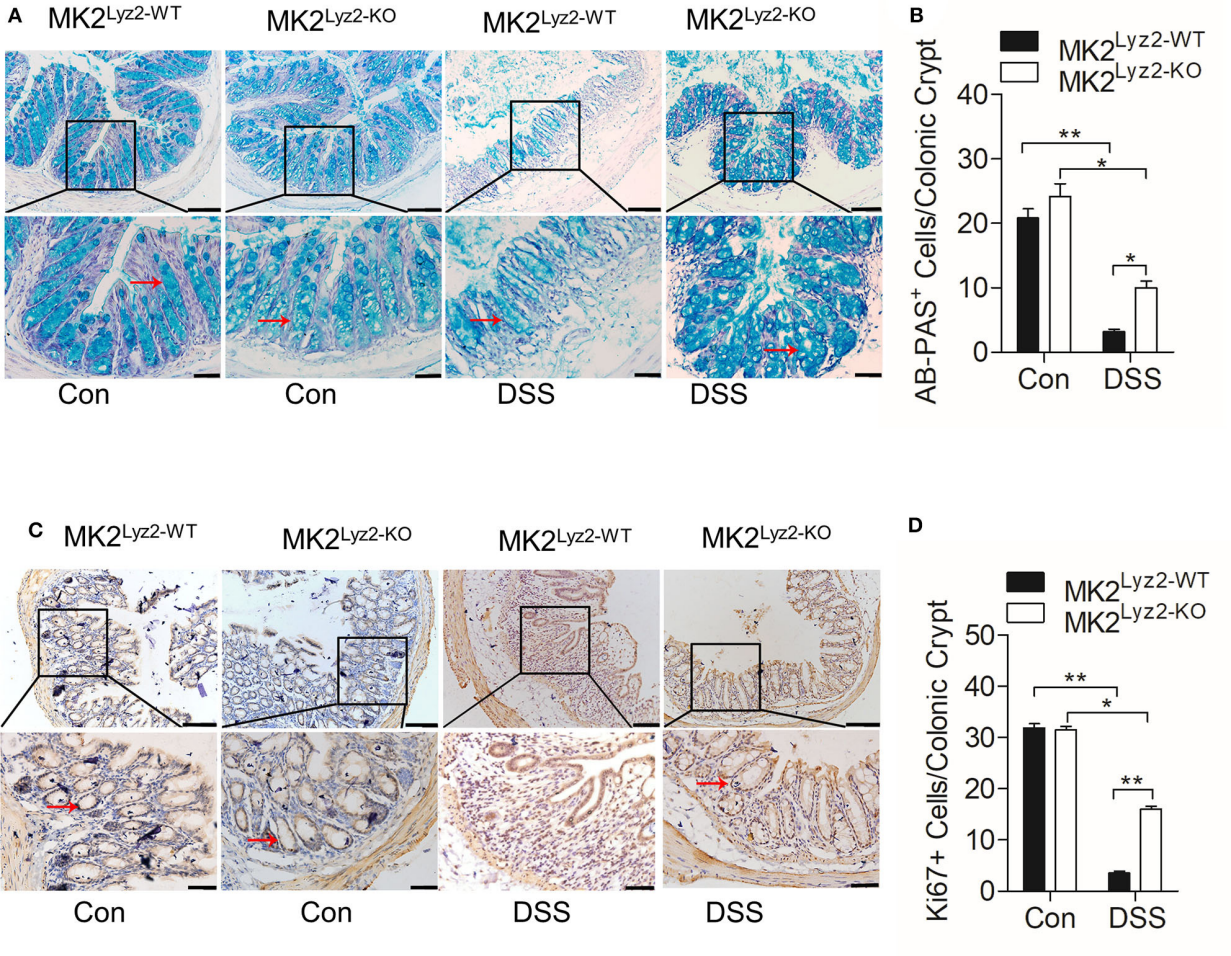
Histological changes of AB-PAS^+^ goblet cells and proliferative cells (Ki67^+^) in MK2^lyz2−WT^ and MK2^lyz2−KO^ mice after 7 days' treatment of 3.5% DSS or water control. **(A)** Representative images showing AB-PAS^+^ goblet cells in colon sections from MK2^lyz2−WT^ and MK2^lyz2−KO^ mice. Scale bars: 100 μm (upper), 40 μm (lower). **(B)** Statistical analysis of AB-PAS^+^ goblet cells in each crypt. **(C)** Representative images showing proliferative cells (Ki67^+^) in colon of DSS treatment mice. Scale bars, 100 μm (upper), 40 μm (lower). **(D)** Statistical analysis of Ki67^+^ cells in each crypt. The results were shown as means ± SEM. *P < 0.05; **P < 0.01 based on six mice in each group.

The integrity of colonic epithelial barrier is maintained partly through mucus secreted by goblet cells (26). In the MK2^lyz2−KO^ mice, there was less reduction in the number of AB-PAS goblet cells per crypt compared to MK2^lyz2−KO^ mice (Figures 2A,B). The ability of colonic epithelial cell regeneration also changed as a result of DSS treatment. As shown in Figures 2C,D, the number of crypt progenitor cells, stained positive with the proliferation marker Ki67, was markedly greater in MK2^lyz2−KO^ mice than that in MK2^lyz2−WT^ mice. These data showed that MK2 in myeloid lineage cells could promote DSS-induced goblet cell damage and inhibit cell proliferation.

### MAPK-Activated Protein Kinase 2 Is Involved in the Regulation of Neutrophil-Derived Reactive Oxygen Species Production

**Figure 3:**
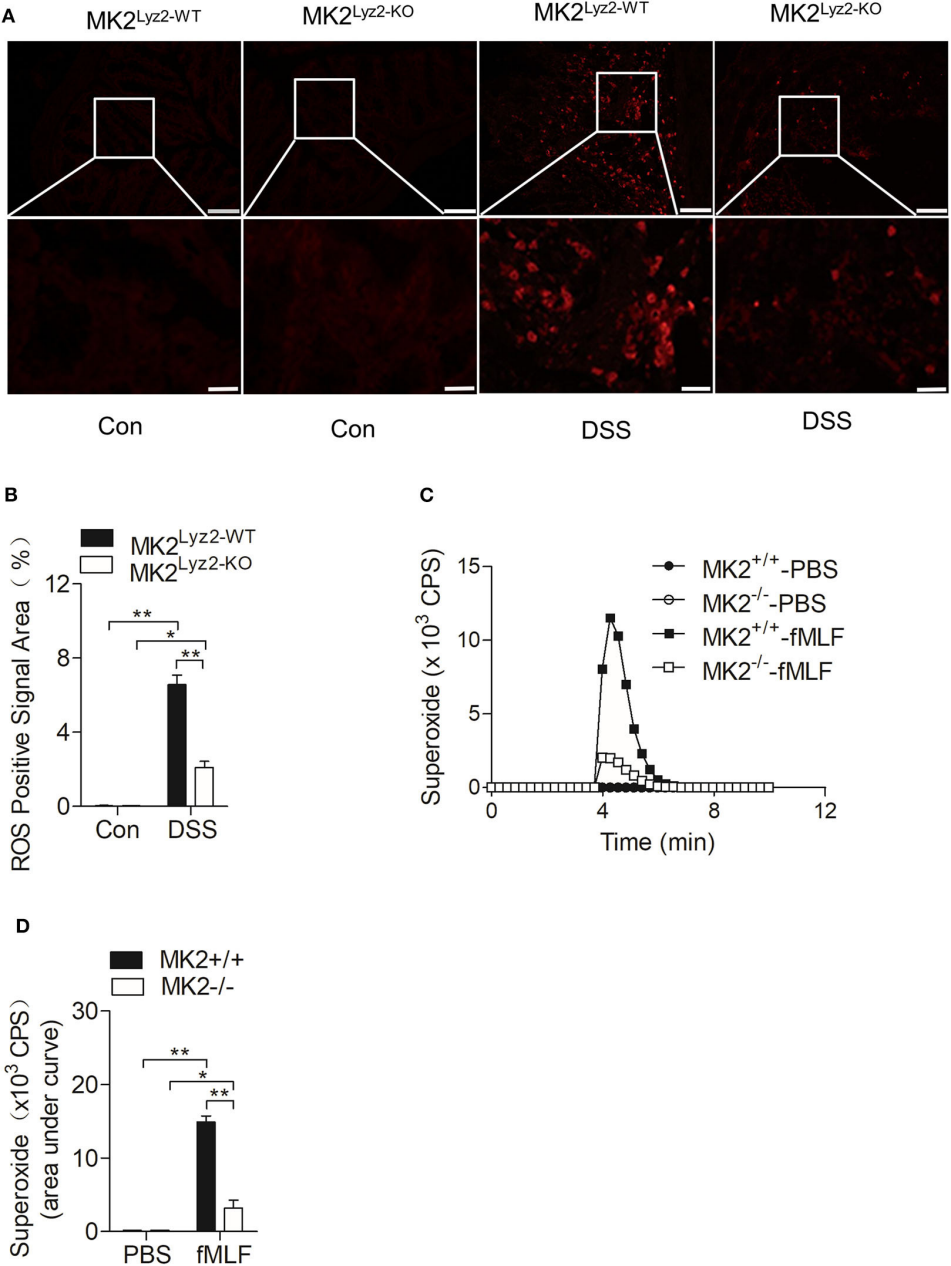
MAPK-activated protein kinase 2 (MK2) involved in the regulation of neutrophil-derived reactive oxygen species (ROS) production. **(A)** Representative images showing ROS-positive signals stained with DCFH-DA on colon sections from MK2^lyz2−WT^ and MK2^lyz2−KO^ mice receiving 3.5% dextran sulfate sodium (DSS) for 7 days. Scale bars, 100 μm (upper), 40 μm (lower). **(B)** Statistical analysis on densitometry of ROS-positive signals to determine the relative ROS level. **(C)** Representative tracing showing the production of superoxide by MK2^lyz2−WT^ or MK2^lyz2−KO^ neutrophils stimulated with 10 μM N-formyl-methionyl-leucyl-phenylalanine (fMLF) or phosphate buffered saline (PBS). Production of superoxide was determined by isoluminol-enhanced chemiluminescence (ECL). **(D)** Statistical of superoxide production. The results were shown as means ± SEM. *P < 0.05; **P < 0.01 based on six mice in each group.

IBD usually accompanied with the elevation of ROS (27, 28), which could lead to persistent inflammation in colon tissues (29). There were many obvious ROS-positive signals in the colon tissues after DSS treatment (Figure 3A), but the ROS-positive signals induced by DSS were significantly reduced in MK2^lyz2−KO^ mice compared to MK2^lyz2−WT^ mice, as indicated by ImageJ software analysis (Figure 3B).

Neutrophils are crucial source of ROS (11). As shown in Figure 1D, there were a large number of leucocytes infiltrated in the colon tissues of MK2^lyz2−WT^ mice after DSS treatment. On the basis of the higher ROS levels in colon tissues of MK2^lyz2−WT^ mice, MK2 might took part in the generation of Neutrophil-derived superoxide. Therefore, we isolated polymorphonuclear neutrophils (PMN) from bone marrow of MK2^lyz2−WT^ and MK2^lyz2−KO^ mice, to determine whether MK2 affected the production of superoxide by PMN. Stimulated by N-formyl-methionyl-leucyl-phenylalanine (fMLF), a PMN chemotactic factor, the superoxide production by PMN from MK2^lyz2−KO^ mice reduced markedly compared to that from MK2^lyz2−WT^ mice (Figures 3C,D). These findings suggested that MK2 took part in the regulation of neutrophil superoxide production.

### MAPK-Activated Protein Kinase 2 Is Required for the Activation of Akt, p38 MAPK, and NADPH Oxidase in Neutrophils

**Figure 4:**
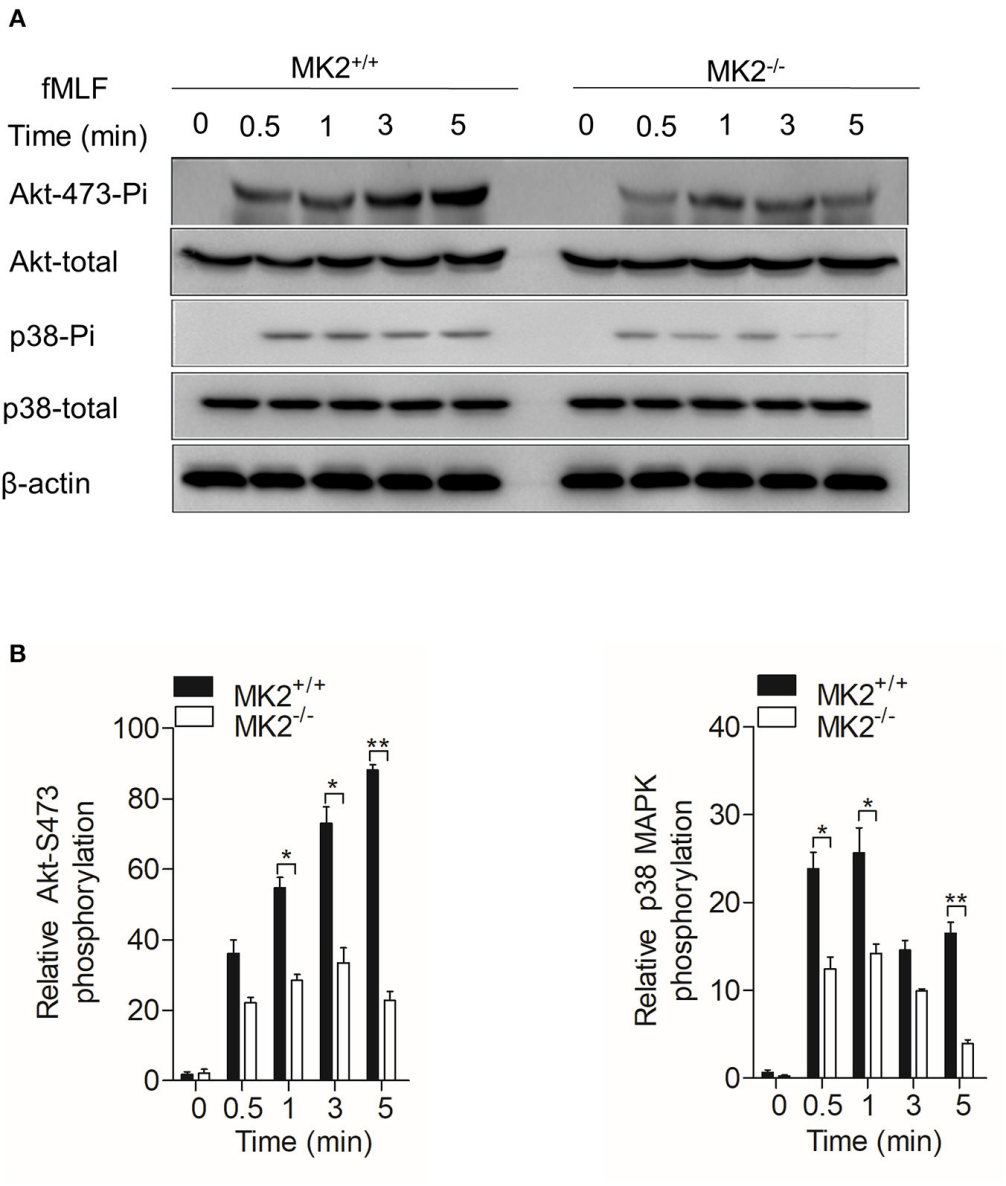
MAPK-activated protein kinase 2 (MK2) was required for Akt and p38 MAPK phosphorylation in neutrophils. Neutrophils from MK2^lyz2−WT^ and MK2^lyz2−KO^ mice were challenged with N-formyl-methionyl-leucyl-phenylalanine (fMLF) (10 μM) for the indicated time. **(A)** Phosphorylation of Akt (Ser473), p38 MAPK were determined by Western blotting using anti-phospho-antibodies against the phospho-Akt (Ser473), total Akt, phospho-p38 MAPK, and total p38 MAPK. **(B)** Densitometry analysis was conducted to determine the relative level of induced Akt phosphorylation and p38 MAPK phosphorylation. Data shown are means ± SEM from three independent experiments. *P < 0.05; **P < 0.01.

In our previous study, we found that MK2 could be activated by p38 MAPK and modulate p47^phox^, which is critical for the formation of the active nicotinamide adenine dinucleotide phosphate (NADPH) oxidase (23, 30). As MK2^lyz2−KO^ neutrophils produced less superoxide when stimulated by fMLF, we therefore detected Akt-p38 MAPK signaling pathways that MK2 had crosstalk with and contributed to the production of superoxide. Neutrophils purified from the MK2^lyz2−KO^ and MK2^lyz2−WT^ mice were stimulated with 10 μM fMLF for 5 min. In response to fMLF stimulation, the phosphorylation of Akt (Ser473) and p38 MAPK were significantly attenuated in MK2^lyz2−KO^ neutrophils, compared to MK2^lyz2−WT^ mice neutrophils, without obviously affecting protein synthesis (Figures 4A,B). Together with our previous findings, these results suggested that MK2 was required for fMLF-induced activation of Akt, p38 MAPK, and NADPH oxidase in neutrophils, and MK2 ablation in the myeloid lineage cells had a negative impact on the activation of Akt, p38 MAPK, and NADPH oxidase, resulting in the reduction of ROS production by neutrophils, therefore alleviating IBD.

## Discussion

In the present research, we found that MK2 modulated neutrophil-derived ROS production and was required for DSS-induced IBD. We showed that MK2 conditional deficiency in myeloid lineage cells markedly alleviated pathological damages and decreased the production of inflammatory cytokines, MPO, and ROS in DSS-induced IBD models by using MK2^lyz2−KO^ mice, in which gene expression of MK2 was abolished in the myeloid lineage cells. We further identified that MK2 deficiency in neutrophils affected ROS production by influencing the activation of Akt, p38 MAPK, and p47^phox^. Therefore, our findings determined that MK2 participated in the overproduction of ROS by neutrophils in the pathogenesis of IBD and which might be based on the activation of Akt, p38 MAPK, and NADPH oxidase by MK2 in neutrophils and suggested that MK2 and ROS might be promising therapeutic targets for IBD.

MK2 as the direct downstream substrate of p38 MAPK is involved in several inflammatory diseases, such as rheumatoid arthritis (RA), psoriasis, vasculitis, and IBD (31). Mourey et al. (32) reported that MK2 contributed to intestinal inflammation in the progression of IBD. Wang et al. reported that MK2 inhibitor could reverse DSS-induced acute colitis by inhibiting the production of a series pro-inflammatory cytokines in the colon and exerted anti-inflammatory effects against inflammatory disease (20, 33, 34). But the specific relationship between MK2 and IBD was not clear and needs to be further explored. Here, in response to DSS challenge, MK2^lyz2−KO^ mice, a mouse strain with MK2 conditional deficiency in myeloid lineage cells, showed an alleviation of colon damage and an inhibition of pro-inflammatory cytokines, MPO, and ROS compared to MK2^lyz2−WT^ mice. These data consistently suggested that MK2 contributes to the pathogenesis of IBD.

MK2 was activated in response to a variety of stimuli, such as oxidative stress, inflammatory cytokines, and DNA damage (35). And the inhibition of MK2 signaling pathway in myeloid cells reduced the expression of inflammatory cytokines, chemokines, ROS, and adhesion factors. The deletion of MK2 leads to decreased immune responses in several inflammatory disease models (36). Our study on MK2^lyz2−KO^ mice showed consistent results that in MK2-deficient myeloid attenuated DSS-induced IBD. In our study, we also found that there were more goblet cells scattered in the intestinal crypt, and the epithelial layer was restored by redistribution of epithelial cells in MK2 conditional mice upon DSS treatment. Together with that more Ki67-positive cells, which showed the cells had higher proliferative ability, were found in the intestinal crypts in MK2 conditional mice, our results suggested that MK2 might regulate epithelial regeneration and repair. Consistently, Henriques et al. (37) found that complete MK2 deletion led to decreased epithelial cell proliferation in the Apc^min/+^ model. On one hand, MK2 regulated pro-inflammatory cytokines, such as IL-6, and has been shown to play an important role in various inflammatory diseases. MK2 in lamina propria myeloid cells might promote intestinal epithelial cell (IEC) proliferation and regeneration at early phase in inflammatory injury *via* inflammatory factor such as IL-6 (38). MK2 has also been shown to control neutrophil migration, and inhibition of p38 MAPK/MK2 activation could attenuate neutrophil respiratory burst activity, exocytosis, chemotaxis, and adhesion (39, 40). Increased neutrophil recruitment intestinal would reduce epithelial proliferation and delay healing of epithelial wounds because of the over adhesion between neuropils and epithelial cell. On the other hand, low concentrations of ROS oxidative stress, which might be produced by PMNs, could drive the cellular responses for repair and regeneration by p38MAPK/MK2 and enhance cell survival and proliferation (41). Thus, compared to wild-type mice that had higher levels of inflammatory cytokines and ROS in injured intestinal tissues, MK2 knockout in myeloid significantly reduced the levels of inflammatory cytokines and ROS at the early phase of IBD in MK2^lyz2−KO^ mice and moderate inflammation not only alleviate tissue injury but also contribute to epithelial proliferation and regeneration.

Neutrophils play a major role in host defense through phagocytosis and production of ROS. When activated, neutrophils produce a mass of ROS. However, the overproduction of ROS by neutrophils *via* NOX2 and MPO could contribute to several diseases (11). Oxidative stress has been proved to be an important part of IBD induced by DSS, and ROS plays a key role to damage intestinal tissues (42, 43). Some studies showed that ROS production was augmented in colonic mucosa of IBD patients (27). IBD patients are characterized by an imbalance between antioxidants and ROS, therefore affecting gut homeostasis (28). Dysfunction of mechanisms that regulate ROS production leads to persistent inflammation and tissue damage (44). And in our study, we identified that MK2 in myeloid lineage cells contributed to the production of ROS in the pathogenies of IBD, causing persist intestinal tissue damage. Furthermore,we proved that MK2 contributes to the pathogenies of IBD by producing superfluous neutrophil-derived ROS when responding to fMLF *in vitro*. During the process of IBD, activated neutrophils are an important source of oxygen-free radicals. Here, using MK2^Lyz2−KO^ mice, our study showed that MK2 contributes to the pathogenies of IBD by producing superfluous neutrophil-derived ROS both *in vivo* and *in vitro*. It is known that knocking out, triggered by Lyz-Cre system, happened not only in PMNs but also in other myeloid cells (45). We should not ignore the possible effects caused by other myeloid cells. As a good case in point, we revealed that mediated by MK2 in neutrophils, IBD was possibly related to neutrophil-derived ROS.

The MAPK family of signaling pathways, consisting of the extracellular signal-regulated kinases (ERKs), Jun N-terminal kinases (JNKs), p38 kinase, ERK3/4, and BMK1 pathways, has interaction with ROS (46). The production of ROS was regulated by JNK and p38 MAPK and ROS, in turn, could induce phosphorylation of ERK1/2 in neutrophils (47). The oxidative stress also could activate p38 MAPK, JNK, and BMK1 signals (46). MK2 is one of the downstream kinases of p38 MAPK and is activated in the process of IBD (20). It is reported that MK2 regulated neutrophil activation by p38-dependent and ERK-dependent signal pathways (39, 48, 49). Rane et al. (50) reported that Akt existed in a signaling complex containing p38 kinase, MK2 and that p38-dependent MK2 activation functions in human neutrophils. Consistently, in our study, we found that phosphorylation of Akt and p38 MAPK was reduced in MK2-deficient neutrophils in response to fMLF stimulation. Our results suggested that the MK2 in myeloid lineage cells, especially neutrophils, contributed to the ROS generation and IBD *via* modulating Akt and p38 MAPK. NOX-2 oxidase, one of the NADPH oxidase (NOX) isoforms, is an inducible electron transport system assembled by different subunits (gp91^phox^, gp22^phox^, p47^phox^, p67^phox^, small GTPase, Rac, and p40^phox^) (51). Phosphorylation of p47^phox^ leads to its membrane translocation that is critical for the formation of the active NADPH oxidase (52). In neutrophils, PMA and fMLF induced MK2 phosphorylation at Ser334 and further increased NADPH oxidase activation (53). Several phosphorylated sites of p47^phox^ are related to NADPH oxidase activation. We previously found that Ser329 of p47^phox^ was a phosphorylation site for MK2 (30). Although we had previously identified that Ser329 of p47phox could be regulated by MK2, the exact effect of these residues on p47^phox^ activation and ROS production should be further explored.

In summary, our studies demonstrated that substantial and significant protection against IBD in MK2 myeloid-specific deletion mice and identified critical roles of neutrophils' MK2 in accentuating intestinal mucosal inflammation through more production of pro-inflammatory cytokines and ROS. In addition, we also found that MK2 played a key role in ROS production through Akt and p38 MAPK signal pathways. Our finding not only revealed the crucial role of neutrophils in IBD but also suggested MK2 and ROS as therapeutic targets for ameliorating IBD.

## Data Availability Statement

The datasets generated for this study are available on request to the corresponding author.

## Ethics Statement

The animal study was reviewed and approved by Institutional Animal Care and Use Committee at Shanghai Jiao Tong University.

## Author Contributions

TZ, JJ, JL, LX, and SD performed the experiments. WZ, LS, and FQ analyzed the data. TZ, JJ, WZ, and FQ prepared the manuscript.

## Conflict of Interest

JJ was employed by the company Shanghai Pharmaceuticals Holding Co. Ltd. The remaining authors declare that the research was conducted in the absence of any commercial or financial relationships that could be construed as a potential conflict of interest.
